# Landscape configuration affects probability of apex predator presence and community structure in experimental metacommunities

**DOI:** 10.1007/s00442-022-05178-9

**Published:** 2022-05-06

**Authors:** Ellie Wolfe, Edd Hammill, Jane Memmott, Christopher F. Clements

**Affiliations:** 1 School of Biological Sciences, University of Bristol, Bristol, BS8 1TQ UK; 2 Department of Watershed Sciences and the Ecology Center, Utah State University, Old Main Hill, Logan, UT USA

**Keywords:** Dispersal, Diversity, Heterogeneity, Protected area, SLOSS

## Abstract

Biodiversity is declining at an unprecedented rate, highlighting the urgent requirement for well-designed protected areas. Design tactics previously proposed to promote biodiversity include enhancing the number, connectivity, and heterogeneity of reserve patches. However, how the importance of these features changes depending on what the conservation objective is remains poorly understood. Here we use experimental landscapes containing ciliate protozoa to investigate how the number and heterogeneity in size of habitat patches, rates of dispersal between neighbouring patches, and mortality risk of dispersal across the non-habitat ‘matrix’ interact to affect a number of diversity measures. We show that increasing the number of patches significantly increases γ diversity and reduces the overall number of extinctions, whilst landscapes with heterogeneous patch sizes have significantly higher γ diversity than those with homogeneous patch sizes. Furthermore, the responses of predators depended on their feeding specialism, with generalist predator presence being highest in a single large patch, whilst specialist predator presence was highest in several-small patches with matrix dispersal. Our evidence emphasises the importance of considering multiple diversity measures to disentangle community responses to patch configuration.

**Supplementary Information:**

The online version contains supplementary material available at 10.1007/s00442-022-05178-9.

## Introduction

Efficient and effective protected areas are needed now more than ever due to the widespread decline of global wildlife populations coupled with an increase in the pressures driving these declines (Butchart et al. 2010). However, protected areas have been criticised for insufficient coverage of biodiverse sites and not meeting the requirements of many taxa (Butchart et al. 2015), prompting recent calls for 30% of land to be conserved by 2030 (CBD 2020) and for nature reserves to be bigger and more connected (Lawton et al. 2010). However, despite being a popular topic for ecological research, the relative importance of the configuration (number of patches and size of patches) and connectivity (levels of dispersal and risk of mortality during dispersal) of landscapes on diversity remains contentious, with recent work demonstrating that the best configuration may depend on the trophic level of the species being conserved (Hammill and Clements 2020).

Reserve design has been a long-running debate in ecology, formalised in the classic question of whether a single large or several small reserve patches (the SLOSS debate; Diamond 1975) are better for biodiversity. Several small reserves are optimal when there is little overlap of species between different patches, meaning more species are supported overall (Bolgovics et al. 2019; Peintinger et al. 2003). In addition, a several-small strategy may be best for biodiversity when several small patches support greater habitat diversity than a single large one (Honnay et al. 1999; MacDonald et al. 2018). Finally, when extinctions are asynchronous across several small patches, rescue effects can occur where dispersal between patches supports populations and enables recolonisation following local extinction (Brown and Kodric-Brown 1977; Holyoak 2000; Hattori and Shibuno 2010). On the other hand, a single large reserve may support larger populations and have lower extinction rates (Burkey 1997; Diamond 1975; MacArthur and Wilson 1967), can support more species when migration between patches is not possible (Liu et al. 2017), allows survival of species with large home ranges, such as large-bodied mammals (Mcnab 1963) and often harbours more rare species than several small patches of the same total size (Jain et al. 2017; Kendal et al. 2017). However, whilst much is known about the relative importance of large and small patches, the effect of both large *and* small patches is rarely considered (Schippers et al. 2009), with previous experimental investigations of habitat configuration considering patches to be homogeneous in size (Burkey 1997; Holyoak 2000). Simulations have shown that landscapes containing varying patch sizes and shapes are more effective in increasing metapopulation survival probability than landscapes containing patches which are all one size (Schippers et al. 2009), but as yet we lack an understanding of the effects of variation in patch sizes in the metacommunity landscape scale. In landscapes with heterogeneous patch sizes, smaller patches can act as predator- or competitor-free refuges (Hattori and Shibuno 2010), meanwhile larger patches support higher population densities (Mccarthy et al. 2011) and better support species with large home ranges (Mcnab 1963). Therefore, we propose that different sized patches hold different communities and consequently heterogeneous landscapes may support higher diversity than homogeneous ones.

Metacommunity theory states that communities in separate habitat patches can be linked by movement between patches which enables regional persistence of a species (Brown and Kodric-Brown 1977; Levins 1969). This suggests that understanding dispersal is of fundamental importance to understanding how diversity can be maintained in multi-patch landscapes. There are many ways in which dispersal enhances diversity—through rescue effects by preventing extinction (Brown and Kodric-Brown 1977), by facilitating evolutionary rescue where movement enables the spread of beneficial mutations which confer adaptation to environmental stress (Bell and Gonzalez 2009), and through spatial insurance whereby dispersal permits movement of species that are adapted to new conditions as a result of environmental change (Loreau et al. 2003).

Conservation practitioners may wish to promote dispersal in nature reserves to prevent extinction. This may be achieved by adding corridors of habitat which directly connect two habitat patches (Gillies et al. 2011; Haddad and Baum 1999; Li et al. 2021), stepping stones which are smaller patches of land in between two larger patches (Fischer and Lindenmayer 2002), or decreasing resistance to movement in the non-habitat matrix surrounding the patches (Gascon et al. 1999). Furthermore, the quality of these connectivity elements can influence their efficacy through changes in dispersal rates and dispersal success. For example, recent work has shown that corridor quality increases not only the probability of individuals dispersing but changes the age-structure of the population in newly colonised patches (Li et al. 2021). Conversely, the potential for poor quality connectivity elements to have a detrimental effect was revealed by a high-resistance matrix increasing the effective isolation of habitat patches, reducing dispersal between them (Ricketts 2001).

The impacts of habitat configuration and connectivity are typically quantified using measures of species richness (MacDonald et al. 2018) or diversity (Bolgovics et al. 2019). However, as the impacts of connectivity and configuration are ultimately governed by species interactions, considering the responses of key species such as predators can further our understanding. For example, unexpected negative effects of corridors can occur when increased predator movement leads to overexploitation of prey (Burkey 1997). Experimental evidence has revealed that corridors can even indirectly benefit predators, where corridors permitting dispersal of prey but not predators lead to increased predator abundance (Limberger and Wickham 2011). In addition, competitive antagonism can drive patterns of patch occupancy. For example, because dominant competitors had high abundances in larger reef patches, inferior competitors occupied smaller patches which they utilised as temporal refuges (Hattori and Shibuno 2010). Furthermore, feeding specialism may also affect species’ responses to habitat configuration and connectivity. Specialists by definition exploit a more limited range of resources than their more generalist counterparts, and consequently suffer more from fragmentation (Tscharntke et al. 2002) and therefore benefit from corridors more (Gillies et al. 2011; Haddad and Baum 1999).

Microcosm experiments using protists are a useful system for investigating the effects of dispersal on diversity within multi-patch landscapes. Protists’ short generation times enable observation of population change over a short period of time, something which would take years using longer-lived organisms (Altermatt et al. 2015). Direct manipulation of dispersal between patches enables consideration of the impact of dispersal rate (Cadotte 2006; Laan and Fox 2019) whilst manipulation of corridors enables consideration of the effects of corridor length (Laurent et al. 2020), matrix harshness (Jacob et al. 2020) and whether patches are connected for predators or prey (Limberger and Wickham 2011). Landscape configuration can be investigated by custom designing multiple landscapes to represent different reserve design scenarios (Hammill and Clements 2020) and populations can be closely monitored throughout the study duration, enabling many metrics to be recorded and compared.

We conducted protist microcosm experiments to investigate the effects of patch-size heterogeneity and dispersal on the SLOSS debate. We utilised custom-designed, 3D-printed landscapes to assess the effects of two features of habitat configuration: the number of patches and patch-size heterogeneity. In addition, we manually manipulated dispersal between patches within a landscape to investigate the effects of local and matrix dispersal, acting as proxies for dispersal through corridors and dispersal across the non-habitat matrix, respectively. To provide a comprehensive overview of the effects of the dispersal events and habitat configuration, we measured population abundances of each species in the system to record a variety of diversity measures: γ diversity, overall number of extinctions, and probability of specialist or generalist predators being present.

## Materials and methods

**Fig. 1:**
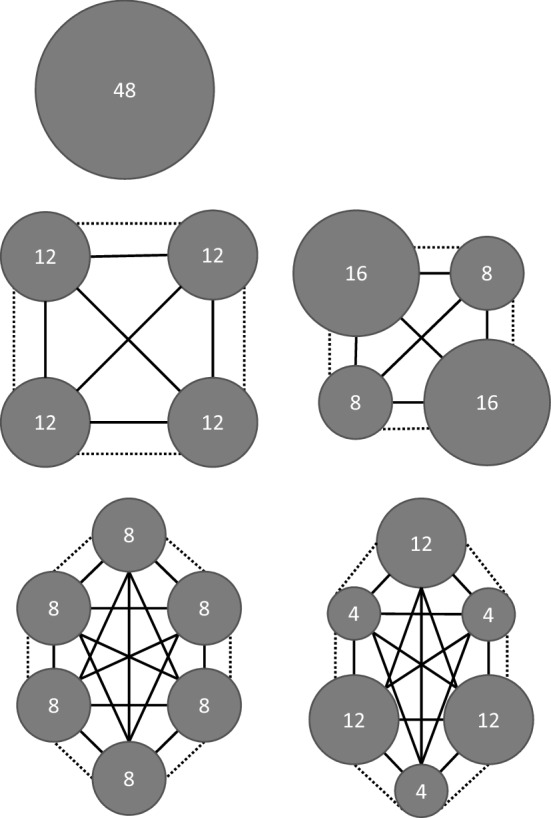
Schematic representation of the five reserve configurations plus dispersal manipulations. Habitat patches (grey circles), their volume in mL (number within grey circles), plus potential local dispersal (dotted lines) and matrix dispersal (solid lines) are all shown. Dotted lines represent one local dispersal ‘event’, and solid lines represent one matrix dispersal ‘event’

To investigate the effects of patch number, patch-size heterogeneity, and dispersal, we manipulated dispersal of protists within microcosms designed to mimic different habitat patch configurations (Figs. 1, S1). The experimental design consisted of five habitat configurations crossed with nine dispersal regimes, resulting in 45 treatment combinations which were replicated four times to total 180 microcosms. The five habitat configurations consisted of one patch, or four or six patches of either homogeneous or heterogeneous patch size (SL, 4Ho, 4He, 6Ho, 6He; Fig. 1). The dispersal regimes consisted of two dispersal types (matrix, M, or local, L) which each had two ‘quality’ levels (low, L, and high, H, mortality risk or low, L, and high, H, frequency, respectively). Due to the time required for sampling, we blocked the replicates by time so that each was sampled on a different day of the week from Monday to Thursday. The experimental landscapes were kept in incubators at a constant temperature of 20 °C for the duration of the 21-day experiment. This equates to approximately 21 generations for the protists as their generation time is around one day (Clements et al. 2013). The patterns in their final abundances were therefore driven by reproduction, extinction, and colonisation within the landscapes.

The protist community consisted of eight protist species, including three apex predators (*Didinium nasutum, Dileptus anser, and Stentor coeruleus*) and five potential prey species (Fig. S2) (all obtained from Sciento, Manchester, UK). *Didinium* and *Dileptus* were considered to be specialists because they fed on two prey species each (Worsfold et al. 2009), whilst *Stentor* was considered as a generalist because it fed on four of the five available prey species (Jiang and Morin 2005; Cadotte et al. 2006; Fig. S2). To comprehensively investigate the effects of the respective predator groups, an ideal experiment would also include treatments with each predator group alone. However, due to time constraints we were unable to include any further treatments in our experimental design.

The experimental landscapes were custom-designed using FreeCAD 3D-design software (Riegel et al. 2020) then 3D-printed using a LulzBot TAZ 6 printer in black PLA filament. To ensure the landscapes were watertight they were then coated in clear epoxy resin. Experimental landscapes consisted of either a single, four, or six circular wells arranged in a ring (Fig. 1). Additionally, wells in landscapes with more than one patch (i.e., four or six patches) were either all the same size (homogeneous) or two different sizes (heterogeneous). In the heterogeneous landscapes we maximised spatial heterogeneity by alternating between small and large patches in the landscape so that each patch was adjacent to patches of a different size. The five reserve configurations (Fig. 1) each had a total volume of 48 ml and were made up of wells with a constant depth of 13 mm to standardise light penetration. Controlling for total volume enabled us to disentangle the effects of heterogeneity and patch number from the effects of landscape area by comparing landscapes with different patch-size distributions. Additionally, we ensured that the average patch size remained constant between homogeneous and heterogeneous landscapes with the same number of patches.

Each landscape was filled with experimental media containing bacteria for the bacterivorous protozoa to feed on, and a carbon source for the bacteria to consume. Experimental media was created by dissolving crushed protozoa pellets (Blades Biological LTD, UK) in spring water (Tesco Ashbeck English natural mineral water) at a concentration of 0.5 g/L and then autoclaving this mixture. On day 5 each bottle of media was inoculated with three bacteria species (*Bacillus subtilis*, *Micrococcus luteus*, and *Pseudomonas fluorescens*). This provided sufficient time for the bacteria to increase to levels which would sustain the protist communities which were added at the start of the experiment on day 0.

We created the experimental community by mixing the eight species in a sterilised jar at densities determined by preliminary experiments (apex predators 1 ind./ml, all prey apart from *Colpidium striatum* 10 ind./ml, *Colpidium striatum* 100 ind./ml). This mixture plus the experimental media were then distributed to fill every patch in each landscape so that each landscape contained a total of 48 ml protist-containing media.

Dispersal between wells was conducted manually rather than via printed corridors as this allowed us to investigate the effects of ‘quality’ of dispersal events by manipulating both dispersal frequency and risk of mortality during dispersal. The two types of dispersal each had two treatment levels plus a no-dispersal control which was applied to all five landscape types. “Local dispersal” occurred between adjacent habitat patches and acted as a proxy for dispersal through corridors connecting neighbouring patches. Local dispersal was either low or high frequency, representing the fact that corridor quality can influence dispersal frequency (Haddad 1999). “Matrix dispersal” occurred from each patch to any patch in the landscape, acting as a proxy for dispersal across the non-habitat matrix to any patch in the landscape. Matrix dispersal was either low or high mortality risk, representing the role an inhospitable matrix can play in causing mortality to dispersing organisms (Nowicki et al. 2014). We chose to manipulate mortality risk of matrix dispersal, but not local dispersal because, although dispersal through corridors is not risk-free, corridors are often created to provide a relatively safer route for passing wildlife, for example when over- and under-passes are added to roads (Simpson et al. 2016). To investigate if there was an interactive effect between the two dispersal manipulations, the two local dispersal manipulations plus control, and two matrix dispersal manipulations plus control were factorially crossed, leading to nine possible dispersal regimes. We chose to factorially cross the dispersal manipulations as previous work has revealed interactive effects between dispersal through corridors and dispersal across the matrix (Åström and Pärt 2013) and we wished to further disentangle this. The treatment levels of the dispersal manipulations were arbitrary as they were designed to demonstrate how general variations in dispersal regime affect diversity outcomes, rather than representing dispersal frequencies or risk of mortality during dispersal for any one taxon.

We conducted dispersal manipulations following sampling and nutrient replenishment. Dispersal occurred weekly for all regimes apart from high frequency local dispersal treatments. Matrix dispersal was conducted by gently mixing a patch through pipetting then removing 0.6 ml from each patch onto a sterile Petri dish. This mixture was then gently mixed and either 0.48 ml (equivalent to 20% mortality, the “low mortality” treatment) or 0.12 ml (equivalent to 80% mortality, the “high mortality” treatment) was pipetted back into every patch (Fig. 1). The remaining solution was discarded, representing the mortality risk associated with dispersing across the matrix. Every patch was topped up with 0.12 ml (low mortality) or 0.48 ml (high mortality) of sterile nutrient medium to account for the volume discarded. This dispersal mode meant that a protist may move into any patch including returning to their initial patch (Fig. 1).

We conducted local dispersal by gently mixing and then pipetting 1.2 ml from every patch onto a sterile petri dish labelled with the initial patch location. 0.6 ml from the 1.2 ml droplet was pipetted into both patches adjacent to the original, transferring protists to their neighbouring patches (Fig. 1). This occurred weekly for the low frequency local dispersal, and twice weekly for high frequency local dispersal, on the sampling day and then again three days later. When a dispersal regime consisted of both local and matrix dispersal, all the media to be dispersed was removed from the landscape prior to pipetting any back, ensuring that no protists were dispersed more than once. This approach to dispersal meant that the dispersal rate was slightly lower for matrix dispersal than local dispersal, as a small proportion of the 0.6 mL taken from a patch in the matrix dispersal treatment was returned to the original patch.

We conducted sampling once a week for each treatment block. Sampling involved searching a well with a microscope for a maximum of five minutes and recording presence or absence of each species. A species was marked as present when at least one individual of the species was spotted. On day 21, the final sampling day, we calculated the abundance of each species by counting individuals in a 0.5 ml subsample taken from each well and multiplying this value according to the well’s total volume. We pipetted up and down within a well to ensure a roughly homogenous distribution of individuals, thus minimising potential bias caused by aggregation of individuals, then pipetted the 0.5 ml subsample onto a sterile petri dish for counting (as in Clements et al. 2013). If a species which was not known to be extinct appeared absent from the subsample, we then checked the whole well and counted all individuals of that species within the well. Although 0.5 ml is a small volume relative to the total volume of the larger wells, this was the maximum amount we could sample in the available time, in particular due to high densities of certain species (>100 individuals in a single subsample).

Following weekly sampling, we conducted nutrient replenishment by mixing each well, removing 10% of each well’s total volume and replacing this with fresh sterile nutrient medium to fill the well to its original volume. This prevented build-up of waste materials and compensated for any evaporation which may have occurred.

### Statistical analyses

The following parameters were used as descriptive variables in our analyses: number of patches (one, four, or six), patch-size heterogeneity (homogeneous or heterogeneous), matrix dispersal (none, low mortality, or high mortality), and local dispersal (none, low frequency, or high frequency). Using data from presence / absence sampling, we calculated the overall number of extinctions in a landscape and probability of specialist (*Didinium nasutum* and *Dileptus anser*) and generalist (*Stentor coeruleus*) apex predator presence. Using the final population count data, we calculated Shannon diversity of each landscape to measure γ diversity. This was calculated using the ‘entropart’ package in R (Marcon and Hérault 2015).

To investigate the relationship between the predictor variables and the response variables, we conducted analyses using Generalised Linear Models (GLMs). The γ diversity GLM had a Gaussian error distribution, specialist and generalist predator presence probabilities had a binomial error distribution, and extinctions had a Quasi-Poisson error distribution due to underdispersion. The saturated model included all descriptive variables and their two-way interactions. To select the best model for each response variable we used Akaike’s Information Criterion corrected for small sample size (AICc, Burnham and Anderson 2002) which ranks models with different parameter combinations by ∆AICc, selecting the ‘best’ model with the lowest AICc. Model simplification was conducted using the ‘dredge’ function from the MuMIn package in R (Bartón 2020) which ranks models according to their AICc values. All models within ∆AICc ≤ 2 of the top model were considered equivalent in their descriptive ability. These top models were subsequently averaged using the ‘model.avg’ function in MuMIn, producing coefficients which were extracted and used for plotting and parameter reporting. In addition to reporting *P* values and effect sizes, we used hierarchical partitioning (Chevan and Sutherland 1991) to estimate the proportion of total variation in each response variable explained by each descriptive variable, thus demonstrating their importance. This analysis was conducted using the ‘hier.part’ package in R (Walsh and MacNally 2020). Furthermore, to investigate the effect of probability of generalist predator presence on probability of specialist predator presence at the patch level we performed a logistic regression (GLM with binomial error distribution). All analyses were conducted in R (version 3.6.3, R Development Core Team 2020).

## Results

### Landscape configuration

**Fig. 2:**
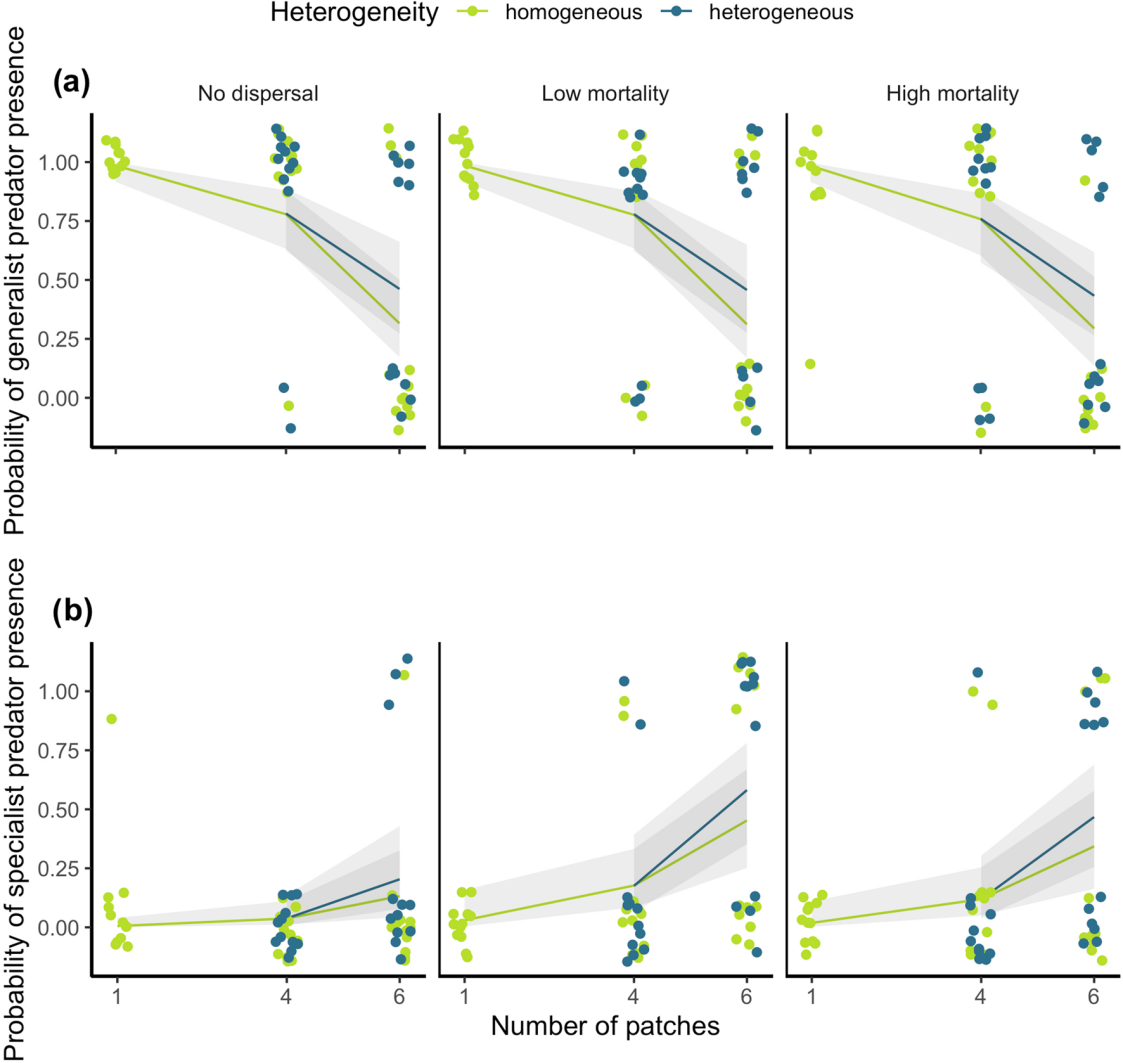
Effects of heterogeneity and number of patches in an ecosystem for each matrix dispersal regime on the probability of a landscape containing **a** generalist predators and **b** specialist predators. Lines are model-averaged binomial GLM outputs, shaded bands are 95% confidence intervals, and points are observed data points (*N* = 12 for each treatment except 4HoHM (*N* = 11), 6HoHM (*N* = 10), and 6HeN (*N* = 11) due to leaking microcosms)

**Fig. 3:**
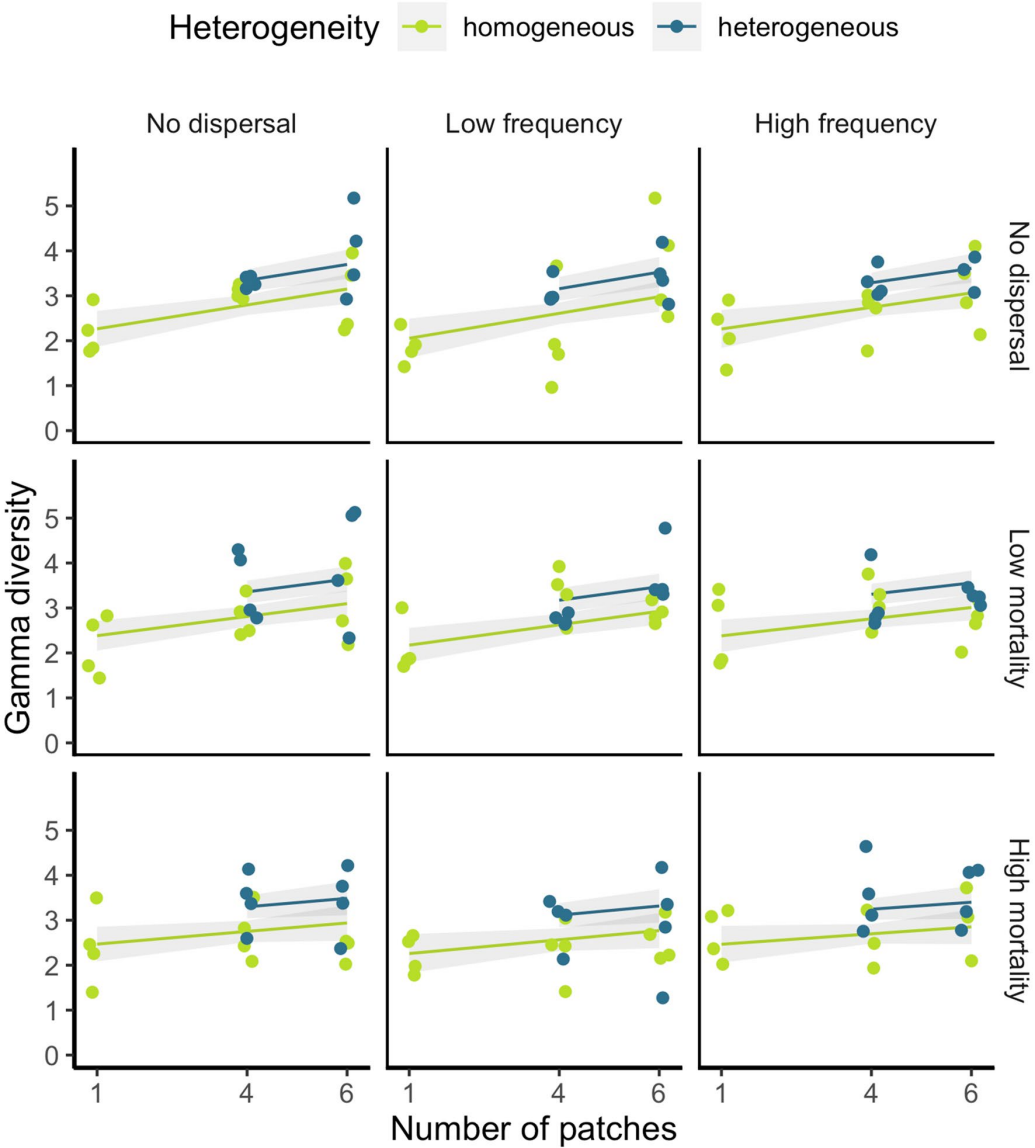
The effects of heterogeneity and number of patches for each dispersal regime on γ diversity. Columns are local dispersal quality level and rows are matrix dispersal quality level. Lines are model-averaged Gaussian GLM coefficients from the top models (∆AICc > 2), shaded polygons are 95% confidence intervals, and points are the observed data points (*N* = 4 for each treatment except 4HoHLHM, 6HoNLHM, 6HoHLHM, 6HeHLNM (all *N* = 3) due to leaking microcosms)

**Fig. 4:**
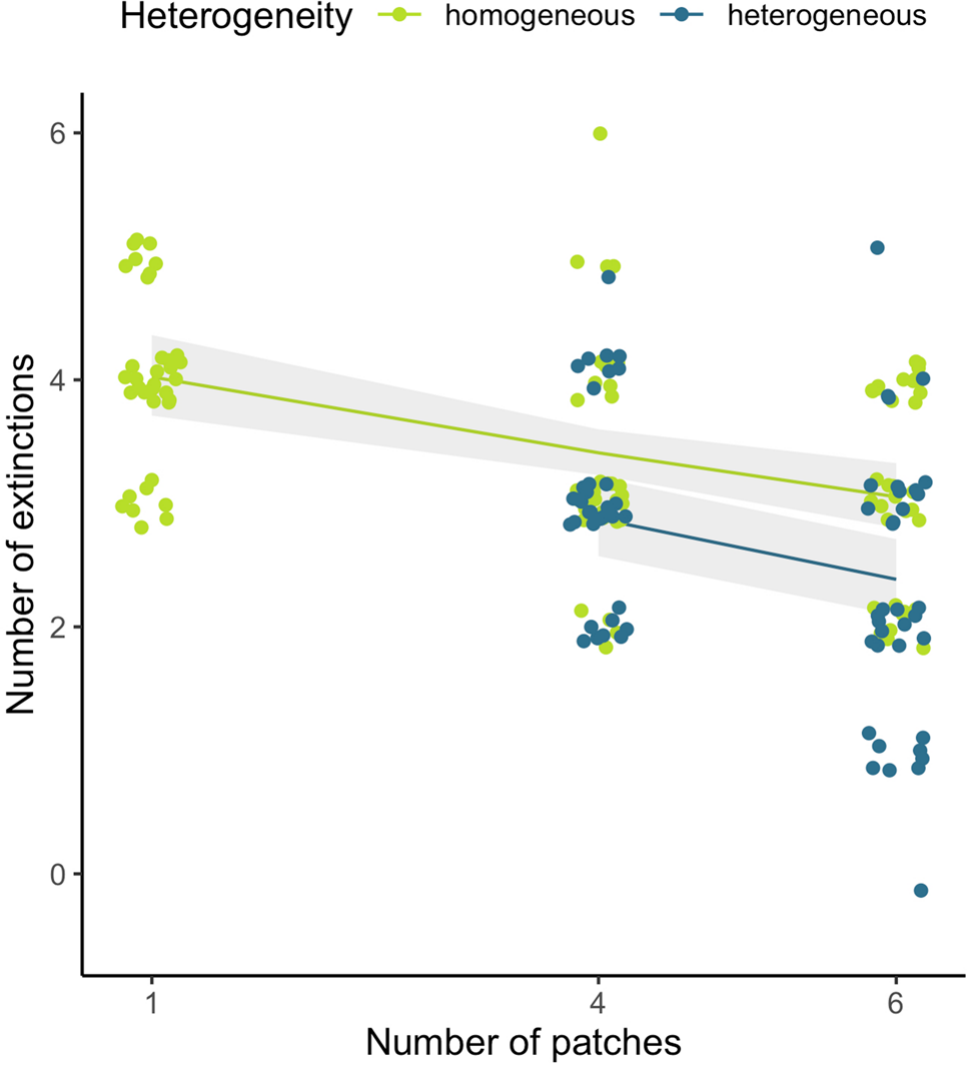
Effect of heterogeneity and number of patches in an ecosystem on overall number of extinctions in a landscape. Top models (∆AICc < 2) did not include matrix or local dispersal as explanatory variables so they are not presented here. Lines are model-averaged Quasi-Poisson GLM model outputs, shaded polygons are 95% confidence intervals, and points are observed data points (*N* = 36 for each treatment except 4Ho (*N* = 35), 6Ho (*N* = 34), and 6He (*N* = 35) due to leaking microcosms)

Increasing the number of patches in a landscape positively influenced three of the diversity measures considered. Increasing the number of patches increased the probability a landscape contained specialist predators (65% variance explained) and increased γ diversity (43.63% variance explained) (Table 1; Figs. 2b, 3). Patch number was therefore the most important predictor of the probability of specialist predator presence, accounting for the highest proportion of variance. In addition, increasing the number of patches decreased the number of extinctions in a landscape (58.08% variance explained), reduced the probability a landscape contained generalist predators (94.13% variance explained), and was the most important predictor of these variables (Table 1; Figs. 2a, 4).

**Table 1:** Model-averaged coefficients ± standard errors (SE) from models explaining specialist and generalist predator presence probabilities, overall number of extinctions, and γ diversity

	Extinctions	Predator presence		γ diversity
		Generalist	Specialists	
Intercept	1.447 ± 0.052 ***	5.359 ± 1.229 ***	5.945 ± 1.269 ***	2.088 ± 0.254***
Patch number	−0.055 ± 0.013 ***	−1.024 ± 0.242***	0.674 ± 0.229**	0.177 ± 0.059 **
Heterogeneity	−0.017 ± 0.252	−1.195 ± 1.903	−1.130 ± 2.188	0.547 ± 0.109 ***
Matrix dispersal (low mortality)		−0.018 ± 0.191	1.709 ± 0.592*	0.153 ± 0.262
Matrix dispersal (high mortality)		−0.117 ± 0.325	1.244 ± 0.607 *	0.286 ± 0.364
Local dispersal (low frequency)				−0.218 ± 0.227
Local dispersal (high frequency)				0.013 ± 0.204
Patch number: Heterogeneity	−0.039 ± 0.052		0.276 ± 0.427	
Patch number: Matrix dispersal (low mortality)				−0.034 ± 0.058
Patch number: Matrix dispersal (high mortality)				−0.083 ± 0.098
Patch number: Local dispersal (low frequency)				0.008 ± 0.034
Patch number: Local dispersal (high frequency)				−0.017 ± 0.044

Any interactions which did not appear in the top models are excluded from the table

* *p* < 0.05; ** *p* < 0.01; *** *p* < 0.001

Heterogeneity did not significantly affect the overall number of extinctions, or the probability of specialist or generalist predator presence. Heterogeneous landscapes had significantly higher γ diversity than homogeneous landscapes (Table 1; Fig. 3), and hierarchical partitioning revealed that heterogeneity was the most important explanatory variable for γ diversity (47.87% variance explained).

Further logistic regression analysis revealed a significant positive effect of patch size on generalist predator presence in heterogeneous landscapes (*P* ≤ 0.05, Fig. S3).

### Dispersal

The probability that a landscape contained specialist predators was significantly increased by matrix dispersal at both low and high mortality levels (Table 1; Fig. 2b), accounting for 25.29% of the variation in the data. However, whilst matrix dispersal had significant effects on the probability of specialist predator being present, it had no effect on the probability of generalist predator presence (Table 1; Fig. 2a).

Unlike matrix dispersal, local dispersal showed no significant main effects on the presence of predators, the number of extinctions, or γ diversity, although local dispersal was included in the top model and explained 6.57% of the variation in γ diversity (Table 1; Fig. 3).

### α and β diversity

We also assessed the effects of our descriptor variables on α and β diversity, revealing a significant positive effect of patch number and heterogeneity on α diversity (Table S1, Fig. S4). Additionally, there was a significant negative effect of matrix dispersal on β diversity at low, but not high mortality levels (Table S1, Fig. S5). We also revealed a significant negative interaction between patch number and matrix dispersal, where at low or no mortality, increasing the number of patches had a positive effect on α diversity, but at high mortality, increasing the number of patches had a negative effect on α diversity (Table S1, Fig. S4).

### Predator coexistence

**Fig. 5:**
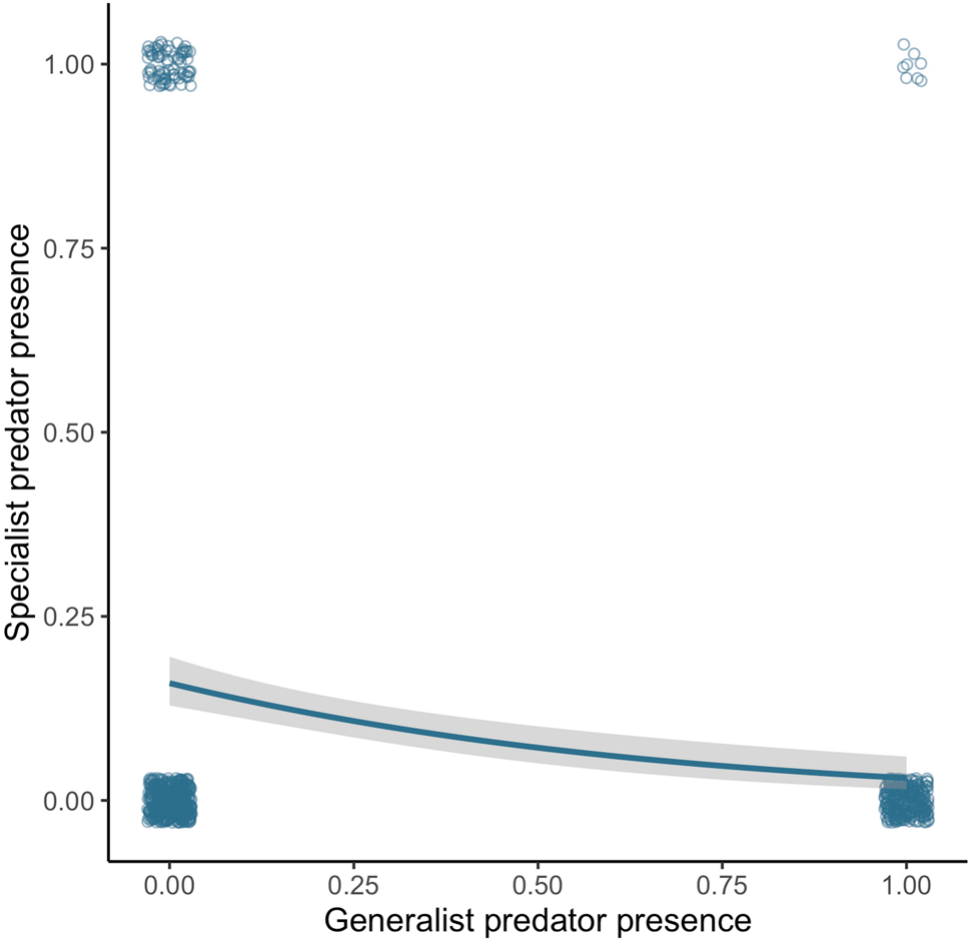
Probability of specialist predator presence as a function of probability of generalist predator presence. Fitted line is the logistic regression, shaded polygon shows 95% confidence intervals, and points are observed data points, jittered slightly for clarity. Relationship was significant (*P* = 0.0000023; *N* = 734)

Analysing data from the patch level revealed that the generalist predator had a significant negative effect on the presence of the specialist predator in our system (*P* ≤ 0.05, Fig. 5).

## Discussion

Understanding the potential trade-offs associated with designing nature reserves is vital if we are to effectively conserve those species most at risk. We investigated experimentally how two key elements of reserve configuration—number of patches and patch-size heterogeneity—interact with two types of dispersal—local and across the matrix—to alter species diversity. Our results revealed that increasing the number of habitat patches can have significant positive effects not just in terms of increased diversity, but also reduced extinctions and increased probability of specialist predator presence. Interestingly, this pattern was reversed for the generalist predator, whose presence was highest in a single large habitat patch. Differences between specialist and generalist predators were further seen in their responses to dispersal, with generalists being unaffected by dispersal whilst specialist predator presence was significantly increased by matrix dispersal, even at high mortality levels. Our results reveal no significant effects of local dispersal on any of the diversity measures considered. Finally, patch-size heterogeneity was the most important predictor of, and significantly increased, γ diversity.

As with previous work (Bolgovics et al. 2019; MacDonald et al. 2018), we find evidence that several small habitat patches maintain higher diversity than a single large habitat patch of the same total area. This reinforces the notion that increasing the number of habitat patches may better promote biodiversity than increasing the area of a single habitat patch (Hammill and Clements 2020). Furthermore, we show that several small patches have fewer extinctions than a single large patch, a finding which is supported by recent evidence (Hammill and Clements 2020), but runs contrary to the traditional belief that larger patches have lower extinction rates (MacArthur and Wilson 1967).

However, whilst increasing the number of patches increased the probability of specialist predator presence, it decreased the probability of generalist predator presence (Fig. 2a, b). This counters the perception that specialists are more sensitive to fragmentation than generalists (dos Anjos et al. 2019) and consequently occur more frequently in larger reserves (Bartonova et al. 2016). As the more-individuals hypothesis states that more productive sites support higher abundances of individuals in a community, thus increasing species presence (Srivastava and Lawton 1998), the reduced presence of the generalist predator *Stentor coeruleus* in the several-small landscapes may be explained by its resource requirements not being met. Apex predators often have large energy requirements and large home ranges (Mcnab 1963), and *Stentor* likely reflects this pattern, requiring more resources due to its body size (~10 times larger than *Didinium nasutum* (Hewett 1988; Jiang and Morin 2005)). It can, therefore, be proposed that the smaller patches were less productive due to their size and were unable to support viable populations of *Stentor.* Furthermore, the specialist predators’ increased presence in the several small landscapes may also be explained by their body sizes. Predators, such as the specialists in our study with smaller body sizes have lower per capita effects on their prey (Emmerson and Raffaeli 2004), and are consequently less likely to over-exploit their prey, increasing their persistence in the smaller patches.

Indeed, this loss of *Stentor* may be an explanatory factor in the patterns observed in the presence of specialist predators: *Stentor* is a voracious generalist predator, known to over-exploit prey and reduce local richness (Cadotte et al. 2006), and therefore may be competitively superior to the specialist predators, reducing their survival when present. We provide evidence that this occurred at the patch level, where there was a negative relationship between probability of generalist predator presence and probability of specialist predator presence (Fig. 5). However, *Stentor’s* resource requirements are not met in the smaller patches, reducing its presence, lessening the competitive antagonism, and increasing the probability of the specialist predators being present in the several-small landscapes. Furthermore, *Stentor* is likely responsible for much of the patterns seen in the diversity responses to landscape design too, particularly that a single large patch had lower γ diversity and more extinctions than the several small patches, as *Stentor* survives best and thus reduces local richness in the single large landscapes (Cadotte et al. 2006). Increasing the number of patches therefore provides positive outcomes for a number of different diversity measures; however, the differences between the responses of generalist and specialist predators highlights the importance of remembering that species interactions ultimately govern community structure at the local scale.

By directly manipulating patch size, we show heterogeneity to be the most important predictor of γ diversity, significantly increasing diversity at the landscape scale. This complements previous simulation modelling demonstrating that metapopulations have a higher survival probability in landscapes containing a mixture of small, large, and linear patches than those containing homogeneous patches (Schippers et al. 2009). Schippers et al. (2009) proposed that this was beneficial because large patches were able to support larger populations, reducing extinction risk, whilst small patches promoted overall landscape connectivity. In the experiments presented here we propose this pattern reflects differences in the specialist and generalist predators: larger patches better meet the range requirements of the larger generalist predator (Fig. 2a), whilst the smaller patches better support specialist predators (Fig. 2b) and reduce the predation pressures on the prey, providing a form of refuge. This is supported by research highlighting the role that small patches play in promoting coexistence when antagonists dominate in larger patches (Hattori and Shibuno 2010). This effect of heterogeneity may be explained by a patch-size threshold effect where a heterogeneous landscape contains patches above and below the size threshold for generalist predator persistence. This is supported by a significant positive effect of patch size on generalist predator persistence in heterogeneous landscapes (Fig. S3). Patches below the threshold may exclude the generalist predator, consequently promoting prey and releasing specialist predators from the negative effects imposed by the generalist predator (Fig. 5). Meanwhile, patches above the threshold may support the generalist predator, ultimately increasing landscape-level diversity. Patch-size heterogeneity is potentially an important and overlooked driver of diversity and would benefit from future research on a larger scale.

To obtain the benefits of both small and large patches in a heterogeneous system, species within a metacommunity must be able to move between the patches. In our experiments, local dispersal between adjacent patches surprisingly had no significant effects on any of the diversity outcomes considered. There are two possible explanations for this: dispersal into neighbouring patches had little effect at the local scale, or that the possible advantages of increased dispersal were outweighed by the disadvantages. Previous research has shown dispersal through corridors to have no effect when populations are not declining and, therefore, will not benefit from increased dispersal (Rantalainen et al. 2004). This, however, does not explain the lack of local dispersal effects in the present study because extinctions did occur in patches, implying that immigration may permit rescue effects. Therefore, it is possible that the positive and negative effects of local dispersal outweighed each other. Dispersal can have a positive impact through rescue effects (Brown and Kodric-Brown 1977; Holyoak 2000) and by enhancing local richness (Cadotte et al. 2006; Schuler et al. 2017). However negative effects may arise, for example when dispersal through corridors enables predators to spread across a system and exploit prey (Burkey 1997; Limberger and Wickham 2011). Additionally, excessive dispersal through corridors can homogenise communities within multi-patch landscapes, causing them to effectively operate as one single large patch (Mouquet and Loreau 2003). The combination of advantages and disadvantages has been revealed in previous research where dispersal had positive effects on local richness, but these were diminished by the negative effects of predation on prey richness (Cadotte et al. 2006). In the present study, it is therefore possible that local dispersal had both positive and negative effects, enabling the spread of prey to support populations via rescue effects, but also the spread of predators to competitively exclude one another, or reduce abundance of prey.

Matrix dispersal had significant effects on one of the four diversity outcomes considered—probability of specialist predator presence (Table 1; Fig. 2b). This positive effect of matrix dispersal on specialist, but not generalist, predator presence may be explained by the predators’ respective feeding specialisms. Generalist can exploit multiple different food sources so can transfer to another prey source without moving to a new area if one species becomes diminished. Specialists, on the other hand, have limited prey food options so if these become exploited, they have two options: disperse to a new patch in search of a new food source, or risk starvation. By dispersing across the matrix to enter any patch in the landscape, specialists have a chance of reaching a patch containing their prey food, thus increasing their survival probability (Fig. 2b). This highlights an important finding, that matrix dispersal which gives individuals the opportunity to disperse to one of several patches may be more beneficial than local dispersal only permitting dispersal between adjacent patches.

As with any laboratory experiment, the extent to which we can extrapolate these findings to larger organisms can be questioned. Ultimately, the dynamics of this system are governed by the same factors we see in larger organisms—competition for resources, predation, mortality risk—meaning that although this system is not an accurate representation of larger organisms, the patterns and processes we see are analogous. Therefore, it is believed that protist microcosm experiments provide a valuable bridge between theory and real-world observations (Altermatt et al. 2015).

In conclusion, whilst the number of habitat patches (Bolgovics et al. 2019), heterogeneity of patch size (Schippers et al. 2009), corridor (Schuler et al. 2017), and matrix dispersal (Åström and Pärt 2013) are all known to influence biodiversity, their interactive effects are rarely considered, especially in relation to more than one conservation goal (but see Altermatt and Holyoak 2012). Here, by simultaneously examining the effects of the number of patches, patch-size heterogeneity, local, and matrix dispersal, we are able to—for the first time—disentangle the relative importance of these factors on various measures of biodiversity. We show that matrix dispersal, multiple patches, and heterogeneity of patch size all positively affected one or more measures of diversity in our system, highlighting their potential use in promoting diversity on a larger scale. Our results also demonstrate the opposing responses of specialist and generalist predators to patch number and matrix dispersal, implying that the role of predator feeding specialism may be an important consideration for designing reserves which cater for a broad range of species. Crucially, we highlight that the most important driver of diversity at the landscape scale in our experimental system was patch-size heterogeneity, suggesting that experiments considering habitat patches to be homogeneous in size may miss an important driver of biodiversity.

## Supplementary Information

Below is the link to the electronic supplementary material.

Supplementary file1 (DOCX 4626 KB)

## Data Availability

The data were deposited in a GitHub repository (https://github.com/chrit88/Experimental-data).

## Code availability

The code is available in a GitHub repository (https://github.com/chrit88/Experimental-data).
