# Supplementary material for: Landscape configuration affects probability of apex predator presence and community structure in experimental metacommunities
Source: Oecologia. 2022 May 6;199(1):193–204. doi: 10.1007/s00442-022-05178-9 (PMC9120115; doi:10.1007/s00442-022-05178-9)
Supplement: Supplementary file 1 — Supplementary file1 (DOCX 4626 KB) [file 442_2022_5178_MOESM1_ESM.docx]

**Landscape configuration affects probability of apex predator presence and community structure in experimental metacommunities**

Ellie Wolfe^1^, Edd Hammill^2^, Jane Memmott^1^, Christopher F. Clements^1^

^1^School of Biological Sciences, University of Bristol, Bristol, BS8 1TQ, UK

^2^Department of Watershed Sciences and the Ecology Center, Utah State University, Old Main Hill, Logan, UT, USA.

**Correspondence author:**

Ellie Wolfe

School of Biological Sciences, University of Bristol, Bristol, BS8 1TQ, UK

[ew16091@bristol.ac.uk](mailto:ew16091@bristol.ac.uk)


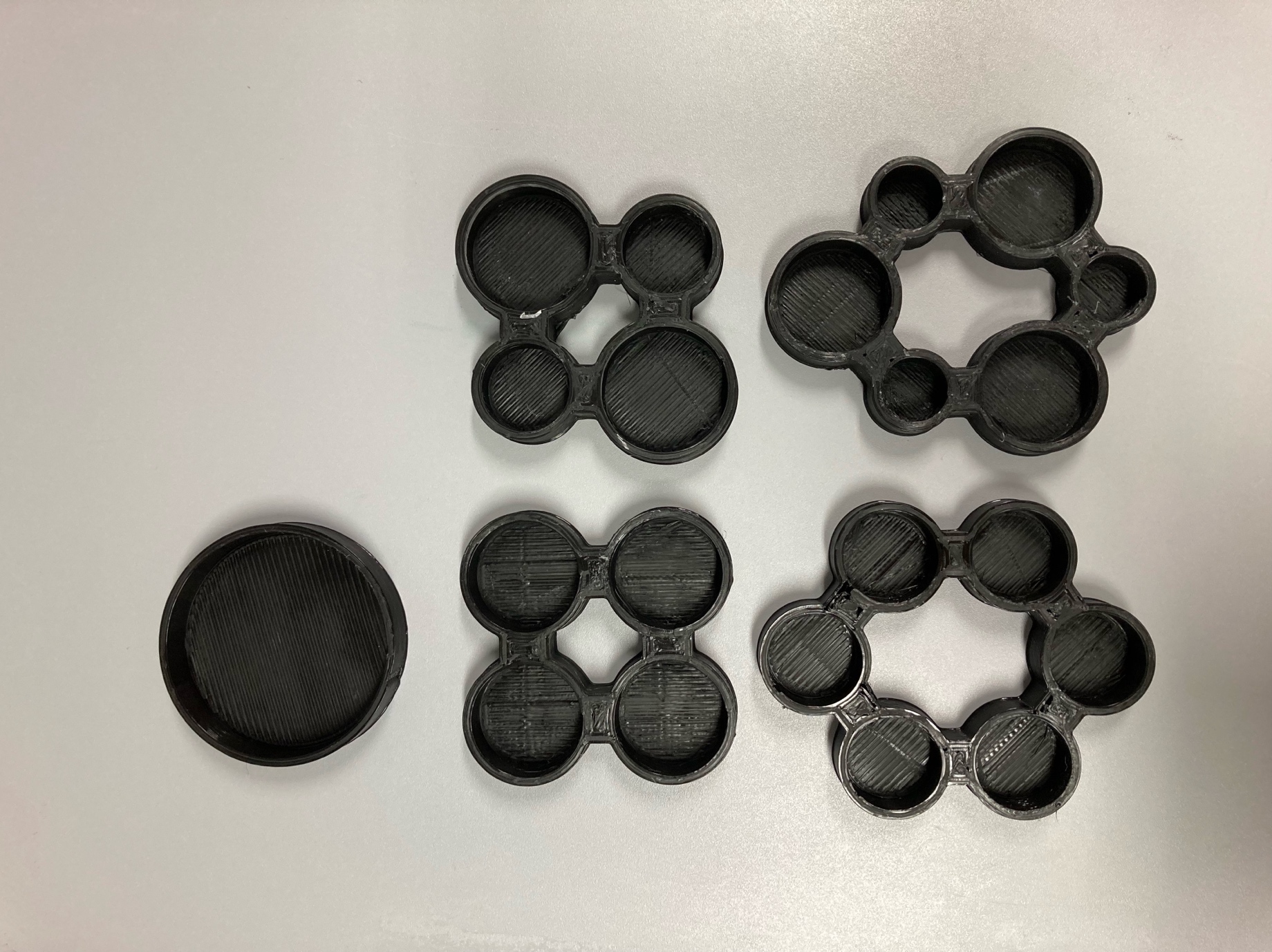
Supplementary materials

**Fig. S1** 3D-printed landscapes that the experiments were conducted in.

**Fig. S2** Food web of the metacommunity used in the experiment, with feeding relationships including predation, intraguild predation, and cannibalism. Arrows represent feeding links, obtained from: (Burkey, 1997; Cadotte et al., 2006; Holyoak, 2000; Kuhlmann & Heckmann, 1994; Warren et al., 2003; Worsfold et al., 2009)

Effect of patch size on generalist predator presence

To further investigate our hypothesis that heterogeneous landscapes had higher γ diversity than homogeneous landscapes because of a threshold effect of patch size on generalist predator presence, we performed a logistic regression (GLM with binomial error distribution) for the heterogeneous landscapes.


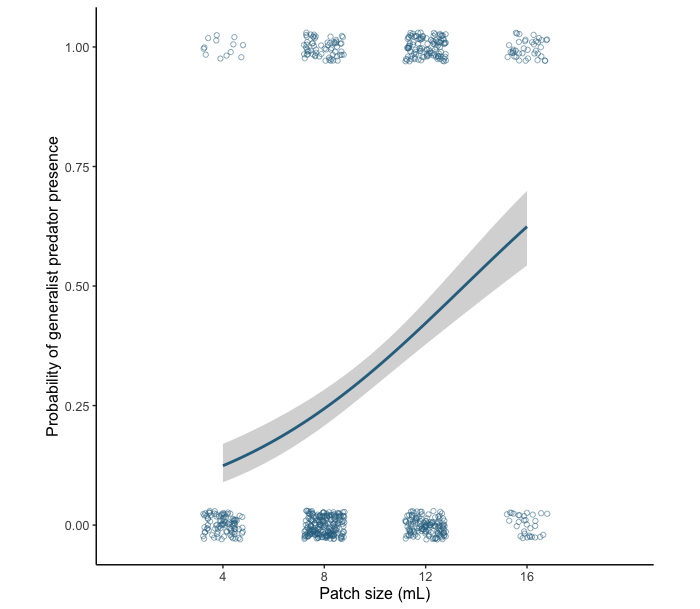


**Fig. S5** The effects of patch size on generalist predator presence in heterogeneous landscapes. Fitted line is the logistic regression, shaded polygon shows 95% confidence intervals, and points are observed data points, jittered slightly for clarity. Relationship was significant (P = 0.00000000000811).

Effect of patch number, patch-size heterogeneity, local dispersal, and matrix dispersal on α and β diversity.

In addition to calculating γ diversity, we also calculated α and β diversity to investigate the effects of our descriptive variables on diversity at the local and between-patch scale. We calculated α and β diversity using the entropart package in R (Marcon and Hérault, 2015), then conducted Generalised Linear Models with a Gaussian error distribution, using the same approach as for γ diversity.


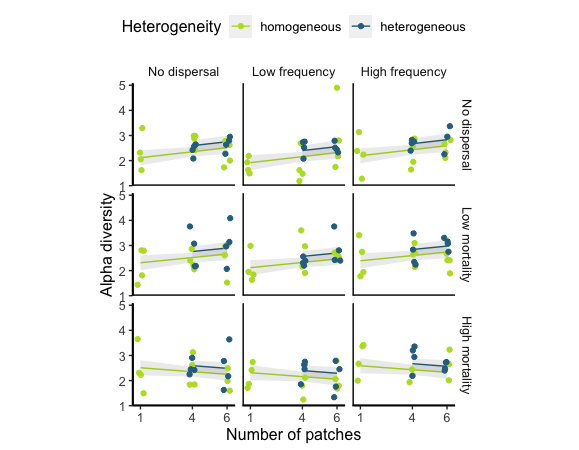


**Fig. S4** The effects of heterogeneity and number of patches for each dispersal regime on α diversity. Columns are local dispersal quality level and rows are matrix dispersal quality level. Lines are model-averaged Gaussian GLM coefficients from the top models (∆AICc > 2), shaded polygons are 95% confidence intervals, and points are the observed data points


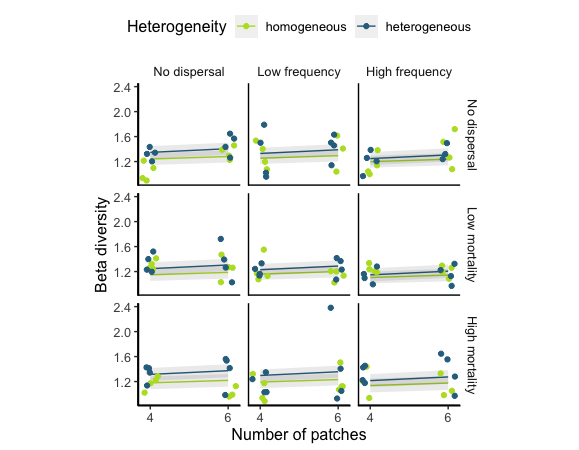


**Fig. S5** The effects of heterogeneity and number of patches for each dispersal regime on β diversity. Columns are local dispersal quality level and rows are matrix dispersal quality level. Lines are model-averaged Gaussian GLM coefficients from the top models (∆AICc > 2), shaded polygons are 95% confidence intervals, and points are the observed data points

**Table S1**. Model-average coefficients ± standard errors from models explaining α and β diversity

|  | α diversity | β diversity |
| --- | --- | --- |
| Intercept | 2.035±0.185*** | 1.162±0.108*** |
| Patch number | 0.079±0.039* | 0.020±0.020 |
| Heterogeneity | 0.240±0.090** | 0.067±0.128 |
| Matrix dispersal (low mortality) | 0.208±0.248 | -0.094±0.042* |
| Matrix dispersal (high mortality) | 0.528±0.248* | -0.062±0.050 |
| Local dispersal (low frequency) | -0.195±0.099 | 0.013±0.046 |
| Local dispersal (high frequency) | 0.079±0.101 | -0.043±0.057 |
| Patch number: Heterogeneity |  | 0.010±0.022 |
| Patch number: Matrix dispersal (low mortality) | -0.011±0.054 |  |
| Patch number: Matrix dispersal (high mortality) | -0.132±0.017* |  |
| Patch number: Local dispersal (low frequency) |  |  |
| Patch number: Local dispersal (high frequency) |  |  |
| Heterogeneity: Matrix dispersal (low mortality) |  | -0.007±0.040 |
| Heterogeneity: Matrix dispersal (high mortality) |  | 0.029±0.062 |
| Heterogeneity: Local dispersal (Low frequency) |  | -0.030±0.059 |
| Heterogeneity: Local dispersal (High frequency) |  | -0.057±0.086 |

**References**

Burkey, T. V. (1997). Metapopulation extinction in fragmented landscapes: Using bacteria and Protozoa communities as model ecosystems. *The American Naturalist*, **150**(5), 568–591. https://doi.org/10.1086/28

Cadotte, M. W., Fortner, A. M., & Fukami, T. (2006). The effects of resource enrichment, dispersal, and predation on local and metacommunity structure. *Oecologia*, **149**(1), 150–157. https://doi.org/10.1007/s00442-006-0426-z

Holyoak, M. (2000). Habitat subdivision causes changes in food web structure. *Ecology Letters*, **3**(6), 509–515. https://doi.org/10.1111/j.1461-0248.2000.00180.x

Kuhlmann, H. W., & Heckmann, K. (1994). Predation risk of typical ovoid and “winged” morphs of Euplotes (Protozoa, Ciliophora). *Hydrobiologia*, **284**(3), 219–227. https://doi.org/10.1007/BF00006691

Warren, P. H., Law, R., & Weatherby, A. J. (2003). Mapping the assembly of protist communities in microcosms. *Ecology*, **84**(4), 1001–1011. https://doi.org/10.1890/0012-9658(2003)084[1001:MTAOPC]2.0.CO;2

Worsfold, N. T., Warren, P. H., & Petchey, O. L. (2009). Context-dependent effects of predator removal from experimental microcosm communities. *Oikos*, **118**(9), 1319–1326. https://doi.org/10.1111/j.1600-0706.2009.17500.x
